# Supplementary material for: Diaci v3.0: chromosome-level assembly, de novo transcriptome, and manual annotation of Diaphorina citri, insect vector of Huanglongbing
Source: Gigascience. 2024 Dec 20;13:giae109. doi: 10.1093/gigascience/giae109 (PMC11659978; doi:10.1093/gigascience/giae109)
Supplement: giae109_Supplemental_Files [file giae109_supplemental_files.zip › Supplemental Figure 1_Wolbachia BRIG plot.docx]

Supplemental Figure 1: A comparison between the two strains of *Wolbachia* and three published genomes.


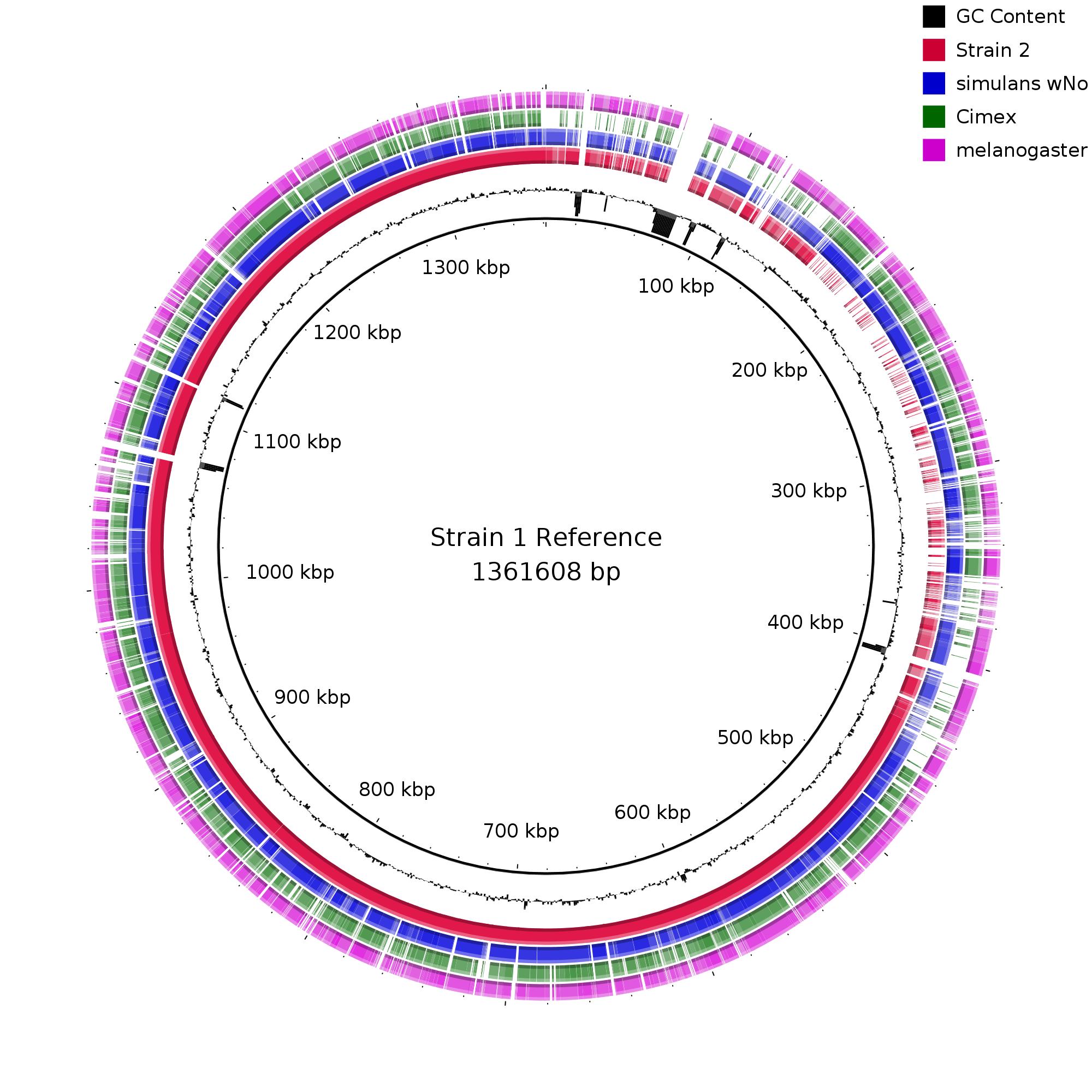


Supplemental Figure 1 Legend: Regions of dark red indicate where strain 1 and strain 2 share the same sequence; where there is more white indicates gaps where strains 1 and 2 differ. Regions of high GC content indicate regions of the reference genome that do not align to the other genomes at all. melanogaster = *Drosophila melanogaster* *Wolbachia* symbiont*,* simulans wNo =  *Drosophila simulans Wolbachia* symbiont, cimex = *Wolbachia* symbiont of *Cimex lectularis*. Accession numbers and references for all *Wolbachia*, *Carsonella* and *Profftella* genomes used for comparison to *D. citri* endosymbionts are shown in the table.

| **Wolbachia Strains** | **Accession Number** | **Reference** |
| --- | --- | --- |
| Wolbachia endosymbiont of Drosophila melanogaster (a-proteobacteria) | ASM802v1 | [(Wu et al. 2004)](https://paperpile.com/c/AGxvRW/Gw4y) |
| Wolbachia endosymbiont strain TRS of Brugia malayi (a-proteobacteria) | ASM838v1 | [(Foster et al. 2005)](https://paperpile.com/c/AGxvRW/csl4) |
| Wolbachia endosymbiont of Onchocerca ochengi (a-proteobacteria) | ASM30688v1 | [(Darby et al. 2012)](https://paperpile.com/c/AGxvRW/Tsva) |
| Wolbachia endosymbiont of Drosophila simulans wNo (a-proteobacteria) | ASM37658v1 | [(Ellegaard et al. 2013)](https://paperpile.com/c/AGxvRW/0QAl) |
| Wolbachia endosymbiont of Cimex lectularius (a-proteobacteria) | ASM82931v1 | [(Nikoh et al. 2014)](https://paperpile.com/c/AGxvRW/TOi4) |
| Wolbachia sp. wRi (a-proteobacteria) | ASM2228v1 | [(Klasson et al. 2009)](https://paperpile.com/c/AGxvRW/GbEN) |
| Wolbachia endosymbiont of Drosophila simulans wHa (a-proteobacteria) | ASM37660v1 | [(Ellegaard et al. 2013)](https://paperpile.com/c/AGxvRW/0QAl) |
| Wolbachia endosymbiont of Onchocerca volvulus str. Cameroon (a-proteobacteria) | W_O_volvulus_Cameroon_v3 | No publication listed* |
| Wolbachia endosymbiont of Drosophila simulans wAu (a-proteobacteria) | Wau001 | [(Sutton et al. 2014)](https://paperpile.com/c/AGxvRW/kAcn)** |
| Wolbachia endosymbiont of Diaphorina citri | wACP3 | [(Saha et al. 2012)](https://paperpile.com/c/AGxvRW/98D4)^ |
| **Carsonella Strains** | **Accession Number** |  |
| Candidatus Carsonella ruddii PV (g-proteobacteria) | ASM1036v1 | [(Nakabachi et al. 2006)](https://paperpile.com/c/AGxvRW/QEEU) |
| Candidatus Carsonella ruddii CE isolate Thao2000 (g-proteobacteria) | ASM28723v1 | [(Sloan and Moran 2012)](https://paperpile.com/c/AGxvRW/FlxZ) |
| Candidatus Carsonella ruddii CS isolate Thao2000 (g-proteobacteria) | ASM28725v1 |  |
| Candidatus Carsonella ruddii HC isolate Thao2000 (g-proteobacteria) | ASM28727v1 |  |
| Candidatus Carsonella ruddii HT isolate Thao2000 (g-proteobacteria) | ASM28729v1 |  |
| Candidatus Carsonella ruddii PC isolate NHV (g-proteobacteria) | ASM28731v1 |  |
| Candidatus Carsonella ruddii DC (g-proteobacteria) | ASM44157v1 | [(Nakabachi et al. 2013)](https://paperpile.com/c/AGxvRW/69jQ) |
| Candidatus Carsonella ruddii (g-proteobacteria) [YCCR] | ASM127451v1 | [(Wu et al. 2015)](https://paperpile.com/c/AGxvRW/248w) |
| Candidatus Carsonella ruddii (g-proteobacteria) [BC] | ASM200935v1 | No publication listed^^ |
| **Profftella Strains** | **Accession Number** |  |
| Candidatus Profftella armatura (b-proteobacteria) | ASM44155v1 | [(Nakabachi et al. 2013)](https://paperpile.com/c/AGxvRW/69jQ) |
| Candidatus Profftella armatura (b-proteobacteria) [YCPA] | ASM127878v1 | [(Wu et al. 2015)](https://paperpile.com/c/AGxvRW/248w) |

*No publication found, but can find the data using this assembly ID and BioProject PRJEB4840 or GenBank GCA_000530755.1

**Can find the data using this assembly ID and BioProject PRJEB6321 or GenBank GCA_000953315.1

^Can find the data using this assembly ID. Could use BioProject PRJNA29451 or GenBank GCA_000331595.1

^^No publication or accession found
