# Supplementary material for: Diaci v3.0: chromosome-level assembly, de novo transcriptome, and manual annotation of Diaphorina citri, insect vector of Huanglongbing
Source: Gigascience. 2024 Dec 20;13:giae109. doi: 10.1093/gigascience/giae109 (PMC11659978; doi:10.1093/gigascience/giae109)
Supplement: giae109_Supplemental_Files [file giae109_supplemental_files.zip › Supplemental Table 1_RNAseq SRA for genome annotation.docx]

Supplemental Table 1: RNA-seq data used for genome annotation and de novo transcriptome assembly.

| SRA | Data | Reference |
| --- | --- | --- |
| SRR1259429 | Citrus spp. CLas- Whole body Adult | [[1]](https://paperpile.com/c/uaVPZl/C7v1x) |
| SRR1259432 | Citrus spp. CLas+ Whole body Adult |  |
| SRR1259461 | Citrus spp. CLas- Whole body Nymph |  |
| SRR1259434 | Citrus spp. CLas+ Whole body Nymph |  |
| SRR2632316 | C. reticulata CLas- Male antennae Adult | [[2]](https://paperpile.com/c/uaVPZl/zhrn7) |
| SRR2632319 | C. reticulata CLas- Female antennae Adult |  |
| SRR2632320 | C. reticulata CLas- Male terminal abdomen Adult |  |
| SRR2632321 | C. reticulata CLas- Female terminal abdomen Adult |  |
| SRR602249 | C. macrophylla CLas- Whole body Adult | [[3]](https://paperpile.com/c/uaVPZl/LEFWa) |
| SRR610529 | C. macrophylla CLas- Whole body Nymph |  |
| SRR610530 | C. macrophylla CLas- Whole body Egg |  |
| SRR5514656 | ACP-Gut-Healthy-rep1a | [[4]](https://paperpile.com/c/uaVPZl/vzjer) |
| SRR5514657 | ACP-Gut-Healthy-rep1b |  |
| SRR5514655 | ACP-Gut-Clas-rep4b |  |
| SRR5514654 | ACP-Gut-Clas-rep4a |  |
| SRR5514651 | ACP-Gut-Clas-rep2b |  |
| SRR5514649 | ACP-Gut-Healthy-rep4b |  |
| SRR5514643 | ACP-Gut-Clas-rep1b |  |
| SRR5514642 | ACP-Gut-Clas-rep1a |  |
| SRR5514653 | ACP-Gut-Clas-rep3b |  |
| SRR5514652 | ACP-Gut-Clas-rep3a |  |
| SRR5514650 | ACP-Gut-Clas-rep2a |  |
| SRR5514648 | ACP-Gut-Healthy-rep4a |  |
| SRR5514647 | ACP-Gut-Healthy-rep3b |  |
| SRR5514646 | ACP-Gut-Healthy-rep3a |  |
| SRR5514645 | ACP-Gut-Healthy-rep2b |  |
| SRR5514644 | ACP-Gut-Healthy-rep2a |  |

References

[1. Vyas M, Fisher TW, He R, Nelson W, Yin G, Cicero JM, et al.. Asian Citrus Psyllid Expression Profiles Suggest Candidatus Liberibacter Asiaticus-Mediated Alteration of Adult Nutrition and Metabolism, and of Nymphal Development and Immunity. *PLoS One*. 2015; doi:](http://paperpile.com/b/uaVPZl/C7v1x) [10.1371/journal.pone.0130328.](http://dx.doi.org/10.1371/journal.pone.0130328.)

[2. Wu Z, Zhang H, Bin S, Chen L, Han Q, Lin J. Antennal and Abdominal Transcriptomes Reveal Chemosensory Genes in the Asian Citrus Psyllid, Diaphorina citri. *PLoS One*. 2016; doi:](http://paperpile.com/b/uaVPZl/zhrn7) [10.1371/journal.pone.0159372.](http://dx.doi.org/10.1371/journal.pone.0159372.)

[3. Reese J, Christenson MK, Leng N, Saha S, Cantarel B, Lindeberg M, et al.. Characterization of the Asian Citrus Psyllid Transcriptome. *J Genomics*. 2014; doi:](http://paperpile.com/b/uaVPZl/LEFWa) [10.7150/jgen.7692.](http://dx.doi.org/10.7150/jgen.7692.)

[4. Kruse A, Fattah-Hosseini S, Saha S, Johnson R, Warwick E, Sturgeon K, et al.. Combining ’omics and microscopy to visualize interactions between the Asian citrus psyllid vector and the Huanglongbing pathogen Candidatus Liberibacter asiaticus in the insect gut. *PLoS One*. 2017; doi:](http://paperpile.com/b/uaVPZl/vzjer) [10.1371/journal.pone.0179531.](http://dx.doi.org/10.1371/journal.pone.0179531.)
