# Supplementary material for: Diaci v3.0: chromosome-level assembly, de novo transcriptome, and manual annotation of Diaphorina citri, insect vector of Huanglongbing
Source: Gigascience. 2024 Dec 20;13:giae109. doi: 10.1093/gigascience/giae109 (PMC11659978; doi:10.1093/gigascience/giae109)
Supplement: giae109_Supplemental_Files [file giae109_supplemental_files.zip › Supplemental Table 2_BUSCO statistics.docx]

Supplemental Table 2: BUSCO statistics.

|  | **BUSCO Arthropoda 1013 Gene Set (%)** | | | |
| --- | --- | --- | --- | --- |
|  | **Complete** | **Duplicated** | **Fragmented** | **Missing** |
| **Diaci v3.0** | 93.5 | 26.4 | 3.3 | 3.2 |
| **Diaci v2.0** | 92.1 | 36.8 | 4.6 | 3.3 |
| **Diaci v1.1** | 82.6 | 4.6 | 11.4 | 6.0 |

|  | **BUSCO Hemiptera 2510 Gene Set (%)** | | | |
| --- | --- | --- | --- | --- |
|  | **Complete** | **Duplicated** | **Fragmented** | **Missing** |
| **Diaci v3.0** | 93.8 | 33.6 | 3.3 | 2.9 |
| **Diaci v2.0** | 93.8 | 33.6 | 3.3 | 2.9 |
| **Diaci v1.1** | 88.9 | 4.7 | 7.0 | 4.1 |

Supplemental Table 2 legend: Percentages of the Arthropoda (arthropoda_odb10) and Hemiptera (hemiptera_odb10) BUSCO gene set that are complete, duplicated, fragmented or missing in Florida *Diaphorina. citri* genome versions, official gene sets and transcriptomes.
