# Supplementary material for: Diaci v3.0: chromosome-level assembly, de novo transcriptome, and manual annotation of Diaphorina citri, insect vector of Huanglongbing
Source: Gigascience. 2024 Dec 20;13:giae109. doi: 10.1093/gigascience/giae109 (PMC11659978; doi:10.1093/gigascience/giae109)
Supplement: giae109_Response_to_Reviewer_Comments_Original_Submission [file giae109_response_to_reviewer_comments_original_submission.pdf]

Author's Response To Reviewer Comments

The reviews are extremely helpful and align well with our thoughts. We would also like to thank the reviewers for providing encouraging feedback.

The edits made to the manuscript in response to the reviewer's comments have been highlighted using track changes.

Reviewer 1
Citrus Greening disease is a massive problem world wide, and its spread by Diaphorina citri has decimated the Florida Orange industry and threatens citrus production around the world.
The authors have generated a modern high quality genome reference and transcript annotation for this infamous vector. The control community has been struggling for far too long with the previous short read based genome reference and low quality annotations that were based on this. Given that control measured based on RNAi, gene drives, population analysis all require a high quality genome, it its critical that this data is released to the public as soon as possible. I am also a huge fan of the curation work, given that all too often too-one looks att he detests they have produced.
I hope that others will follow this example.
The manuscript is well written, the assembly is well done and I commend the authors on doing this right, and persisting for so long to get this genome reference complete.
Given the need for this high quality dataset, I see no reason to delay publication, and suggest it be accepted as is.

We deeply appreciate the comments and the reviewer's recognition of our highly collaborative and community-driven annotation initiative that we started 8 years ago and will continue beyond this publication. We have also given priority to prompt and open sharing of data and resources to the research community.

Reviewer 2
In this manuscript, the authors constructed a high-quality chromosomal level genome of Diaphorina citri based on PacBio long reads followed by proximity ligation-based scaffolding. A 474 Mb genome was obtained and 13 chromosomes were assembled through Hi-C sequencing. The obtained results could provide useful genomic resources for the control of D. citri and Huanglongbing. In general, this work has been well done and the data analysis is solid. However, there are some minor issues that need to be clarified before publication. Detailed comments were found below.
Analyses and Discussion part:
2.1 Genome assembly:
1. Genome assembly version v3.0 may be displayed more intuitively after Circos circular visualization analysis.
We had carefully evaluation multiple options for visualization of the synteny of the ACP genome assembly to the Pachypsylla venusta genome including Circos plots. The finally settled on the parallel plot (Figure 2) for representing the structural comparison of the two genomes on a linear framework as we think this shows the chromosome by chromosome synteny clearly. Adding a Circos plot for the ACP genome with the standard tracks may not offer additional information to the reader beyond what we have provided in Figures 2 and 3.

2. The website citrusgreening.org is not available to be accessed. Some details about the genome cannot be reviewed.
The citrusgreening.org portal is actively used for our annotation projects and is updated periodically. I would like to request the reviewer to try to access it again. If this is the first time visiting the page, please give it a few seconds to load all the libraries and graphics as that may be causing the timeout error.

3. The resolution of Fig. 2 - Fig. 4 is too low. I recommended to replace them with clear figures.
Figures 2 and 3 depict the synteny of the ACP genome to related genome assemblies. We have followed the submission instructions and both images are 300 dpi as required in the submission instructions

https://academic.oup.com/gigascience/pages/instructions_to_authors?login=false#Preparing%20Figures
Is it possible the PDF conversion process is impacting the quality of the image available to the reviewer? We have made a note of this and will be reviewing the proofs carefully if and when we get to that stage. We are including a higher resolution image for Figure 4 at 300 dpi

2.4 De novo Transcriptome
1. I didn't find the file "Supplemental Table 3".
We thank the reviewer for bringing this to our attention. This was an error on our part as the reference should have been to Supplemental Table 1. We have fixed this in the revised article.
2.5 Pathway-based manual curation
1. In this section, the author has done a lot of important pathway analysis, which has great enlightening value for the effective prevention and control of D. citri and Huanglongbing. However, I feel like there is no summary result that can be displayed to the reader, so I suggest adding a table to summarize the findings of each pathway.
This is an excellent idea as it improves the readability of the manuscript and provides a quick summary of a large amount of work. We have added a new Table 5 Summary of pathway-based manual curation results in section 2.5.2 Specific pathways targets for curation.
Table 5. Summary of pathway-based manual curation results

Pathway/Gene Family	Genes Annotated	Reference
Development		
Segmentation	25	67
Wnt signaling	24	54
Hedgehog signaling	13	
Notch signaling	16	
Insulin signaling	5	
Hox genes and cofactors	14	66
Immune Response		
Melanization	12	
Yellow	9	55
Metabolic and Cellular functions		
Carbohydrate metabolism	32	52
Chitin metabolism	19	61,62,68
V-APTases	14	53
Ubiquitin-proteasome pathway	15	51
Chromatin remodeling	27	
Light sensing		
Circadian rhythm	27	50
Phototransduction	21	
Reproduction	20	

Table 5 Legend: Numbers of genes annotated in Diaci v3.0 as part of our pathway-based manual curation approach. References are given for reports published as part of the Asian citrus psyllid community annotation series in Gigabyte.
*Methods part
2. Iso-Seq：
1.The author needs to provide the related information about the carrier rate or level of CLas in the CLas infected population of D. citri.
The healthy and CLas-infected adult (mixed sex pooled) and nymphs (mixed sex 4th and 5th instars pooled) were sourced from colonies reared on Citrus medica 'citron'. The CLas-infected psyllids were raised on Citrus medica 'citron' with a CLas CT value of more than 36. The samples were generated in 2016. We are trying to track down the infection rate of the colony but it has not been possible due to a number of changes in personnel at the Ft. Pierce lab. We hope to get more information in the near future and will add it to the proof if and when we get to that stage.

Please let me know if you have any questions. Thank you for your time.
