# Supplementary material for: Diaci v3.0: chromosome-level assembly, de novo transcriptome, and manual annotation of Diaphorina citri, insect vector of Huanglongbing
Source: Gigascience. 2024 Dec 20;13:giae109. doi: 10.1093/gigascience/giae109 (PMC11659978; doi:10.1093/gigascience/giae109)
Supplement: giae109_Reviewer_2_Report_Original_Submission — Xuefeng Wang -- 4/3/2023 Reviewed [file giae109_reviewer_2_report_original_submission.pdf]

Reviewer Report

Title: Diaci v3.0: Chromosome-level assembly, de novo transcriptome and manual annotation of Diaphorina citri, insect vector of Huanglongbing

Version: Original Submission **Date:** 4/3/2023

Reviewer name: Xuefeng Wang

Reviewer Comments to Author:

In this manuscript, the authors constructed a high-quality chromosomal level genome of Diaphorina citri based on PacBio long reads followed by proximity ligation-based scaffolding. A 474 Mb genome was obtained and 13 chromosomes were assembled through Hi-C sequencing. The obtained results could provide useful genomic resources for the control of D. citri and Huanglongbing. In general, this work has been well done and the data analysis is solid. However, there are some minor issues that need to be clarified before publication. Detailed comments were found below.
Analyses and Discussion part:
2.1 Genome assembly:
1. Genome assembly version v3.0 may be displayed more intuitively after Circos circular visualization anlysis.
2. The website citrusgreening.org is not available to be accessed. Some details about the genome cannot be reviewed.
3. The resolution of Fig. 2 - Fig. 4 is too low. I recommended to replace them with clear figures.
2.4 De novo Transcriptome
1. I didn't find the file "Supplemental Table 3".
2.5 Pathway-based manual curation
1. In this section, the author has done a lot of important pathway analysis, which has great enlightening value for the effective prevention and control of D. citri and Huanglongbing. However, I feel like there is no summary result that can be displayed to the reader, so I suggest adding a table to summarize the findings of each pathway.
*Methods part
2. Iso-Seqï¼š
1.The author needs to provide the related information about the carrier rate or level of CLas in the CLas infected population of D. citri.

Methods

Are the methods appropriate to the aims of the study, are they well described, and are necessary controls included? Choose an item.

Conclusions

Are the conclusions adequately supported by the data shown? Choose an item.

Reporting Standards

Does the manuscript adhere to the journal's guidelines on [minimum standards of reporting?]() Choose an item.

Choose an item.

Statistics

Are you able to assess all statistics in the manuscript, including the appropriateness of statistical tests used? Choose an item.

Quality of Written English

Please indicate the quality of language in the manuscript: Choose an item.

Declaration of Competing Interests

Please complete a declaration of competing interests, considering the following questions:

- Have you in the past five years received reimbursements, fees, funding, or salary from an organisation that may in any way gain or lose financially from the publication of this manuscript, either now or in the future?
- Do you hold any stocks or shares in an organisation that may in any way gain or lose financially from the publication of this manuscript, either now or in the future?
- Do you hold or are you currently applying for any patents relating to the content of the manuscript?
- Have you received reimbursements, fees, funding, or salary from an organization that holds or has applied for patents relating to the content of the manuscript?
- Do you have any other financial competing interests?
- Do you have any non-financial competing interests in relation to this paper?

If you can answer no to all of the above, write 'I declare that I have no competing interests' below. If your reply is yes to any, please give details below.

I declare that I have no competing interests

I agree to the open peer review policy of the journal. I understand that my name will be included on my report to the authors and, if the manuscript is accepted for publication, my named report including any attachments I upload will be posted on the website along with the authors' responses. I agree for my report to be made available under an Open Access Creative Commons CC-BY license (http://creativecommons.org/licenses/by/4.0/). I understand that any comments which I do not wish to be included in my named report can be included as confidential comments to the editors, which will not be published.

Choose an item.

To further support our reviewers, we have joined with Publons, where you can gain additional credit to further highlight your hard work (see: https://publons.com/journal/530/gigascience). On publication of this paper, your review will be automatically added to Publons, you can then choose whether or not to claim your Publons credit. I understand this statement.

Yes Choose an item.
